# Clinical Presentation and Integrated Management of Pressure Injuries in the Emergency Hospital Setting: A Plastic Surgeon’s Perspective

**DOI:** 10.3390/healthcare12242583

**Published:** 2024-12-22

**Authors:** Stefan Cacior, Eliza-Maria Bordeanu-Diaconescu, Vladut-Alin Ratoiu, Andreea Grosu-Bularda, Florin-Vlad Hodea, Khalid Al-Falah, Razvan-Nicolae Teodoreanu, Serban-Arghir Popescu, Ioan Lascar, Cristian-Sorin Hariga

**Affiliations:** 1 Department 11, Discipline Plastic and Reconstructive Surgery, University of Medicine and Pharmacy Carol Davila, 050474 Bucharest, Romania; stefan.cacior@drd.umfcd.ro (S.C.);; 2 Clinic of Plastic Surgery and Reconstructive Microsurgery, Clinical Emergency Hospital of Bucharest, 014461 Bucharest, Romania; 3 Burn Centre, Emergency Clinical Hospital of Bucharest, 014461 Bucharest, Romania

**Keywords:** negative pressure vacuum therapy, perforator flap, musculocutaneous flap, pressure injury, pressure ulcer

## Abstract

**Background**: Pressure injuries are localized areas of tissue damage or necrosis that occur when pressure is applied to the skin for prolonged periods, often over bony prominences, often the sacrum, heels, ischial tuberosities, and greater trochanters. The pathophysiology is complex, involving a combination of mechanical forces, ischemia, and tissue hypoxia. **Methods**: We conducted a 2-year retrospective study aiming to assess the clinical characteristics, risk factors, and management of pressure injuries in patients admitted to an emergency hospital who underwent a plastic surgery examination. **Results**: This study included 176 patients with clinically diagnosed pressure ulcers, with findings showing 28.52% of cases as stage III and 35.57% as stage IV. Common sites included the sacrum (40.94%), ischium (15.1%), and heel (14.43%). The median patient age was 76 years, with 47.15% between 60 and 80 years and 36.93% ≥ 80 years, often presenting with comorbidities increasing the risk of pressure injuries, such as cardiovascular disease (71.59%), diabetes (18.18%) and obesity (9.66%). Important risk factors included neurological diseases (46.02%), spinal cord injuries (14.7%), and nutritional deficiencies, as indicated by anemia (10.43g/dL; 95% CI [10.04; 10.82]), low serum albumin (2.56 g/dL; 95% CI [2.43; 2.69]) and proteins (5.54 g/dL; 95% CI [5.34; 5.73]). Mortality was significant, at 36.93%, with 23.3% occurring within the first 7 days of hospitalization due to the patients’ critical condition. Decision-making for surgical intervention considered the patient’s general status, comorbidities, and ulcer severity. Surgical treatment consisted of seriate debridement, negative pressure vacuum therapy, and/or coverage using skin grafting, local advancement, or rotation flaps. **Conclusions**: The key question for a plastic surgeon to consider is how pressure ulcers should be managed. Various debridement and covering techniques should be tailored to the wound’s characteristics, considering patient comorbidities and general health condition.

## 1. Introduction

Pressure injuries, also known as pressure ulcers, bedsores, pressure sores, or decubitus ulcers, represent a lesion of the skin and underlying tissue as a result of prolonged pressure and associated ischemia, tissue inflammation, and tissue anoxia, developing most often on bony areas of the body, such as the occipital prominence, acromion process, scapula, elbows, thoracic and lumbar spine, sacrum, ischial tuberosities, greater trochanter, knees, medial and lateral malleolus, and heels [1,2].

Pressure sores are commonly observed in patients who are acutely hospitalized or those who need prolonged care in institutional settings; more than 60% of bedsores develop within the hospital words. They are observed especially in older patients, the critically ill, and the spinal cord injury population. Over 50% of patients with pressure ulcers are 70 years or older [3]. In patients with spinal cord injuries (SCI), the reported incidence of pressure ulcers is between 25% and 66% [4]. Other risk factors observed in pressure sores are nutritional deficiency, malnutrition, and hypoalbuminemia, which affect soft tissue integrity and healing, lower body weight, cognitive and physical impairments, urinary or fecal incontinence, diabetes mellitus, vascular insufficiency, contractures, and smoking [5]. External factors such as prolonged pressure, friction, shear forces, and moisture also contribute to the development of pressure injuries [2,6].

Data from the literature consistently shows an inverse relationship between pressure and the time to ulceration, highlighting that the greater the pressure applied, the shorter the time needed to cause tissue damage. Brooks and Duncan, in their pioneering experimental work, demonstrated that higher pressure values require less time to induce irreversible damage to tissues, which is particularly relevant in the context of patients who are immobile or unable to reposition themselves, as they may experience both prolonged and high-pressure exposure in certain body areas [7]. In another experimental study, Dinsdale demonstrated that a constant pressure of 70 mm Hg applied for longer than 2 h could lead to irreversible tissue damage. This threshold pressure was found to be sufficient to compromise blood flow and cause ischemia, eventually leading to cell death and tissue necrosis if sustained over time. However, Dinsdale also observed that intermittent relief of pressure significantly mitigated tissue damage, and even when pressures as high as 240 mm Hg were applied, minimal tissue damage was observed, provided the pressure was periodically released [8].

Skeletal muscle tissue is more vulnerable to pressure damage than skin, likely due to its higher capillary and mitochondrial volume density, which is linked to its increased metabolic demand. In contrast, skin is significantly stiffer than muscle or adipose tissue, causing it to deform less and making it less prone to ischemic damage. In animal studies, the initial signs of ischemic damage in skeletal muscle appear after 2 to 4 h of sustained pressure [4,9]. Histopathological studies have shown that what initially appears as superficial skin erythema is often just the “tip of the iceberg”, concealing more extensive underlying tissue damage. Pressure-induced injury typically begins in deeper structures, such as muscle and subcutaneous fat, where blood flow is more easily compromised due to the greater susceptibility of these tissues to ischemia [10].

The principle “prevention is better than cure”, is particularly true when it comes to pressure ulcers. Prevention involves addressing both extrinsic factors, such as prolonged pressure, friction, shear forces, and moisture, and intrinsic factors, including nutrition, mobility, and comorbidities. One of the most effective measures is frequent repositioning of patients, particularly those who are immobile or bedridden, to avoid prolonged pressure on high-risk areas of the body, such as the sacrum and the heels. Regular repositioning helps distribute weight more evenly, reducing the likelihood of tissue ischemia. Along with repositioning, maintaining good nutrition is essential to support skin health and overall healing. The use of specialized bedding, such as pressure-relieving mattresses and cushions, can further reduce pressure on sensitive areas, providing additional comfort and protection [11,12,13]. Once a pressure injury appears, therapeutic management ranges from topical agents for debridement and infection control to therapeutic agents that promote wound healing. Additional management strategies include nutritional support to enhance tissue repair, regular repositioning to reduce pressure, the use of special mattresses and cushions to offload pressure, and surgical interventions when necessary [14,15].

Primary prevention of pressure injuries focuses on proactive strategies to minimize the risk of skin breakdown due to prolonged pressure on vulnerable areas of the body.

Surgical treatment is usually considered in cases where conservative has failed or when the ulcer has progressed to a severe stage. Surgical intervention is most commonly required for large, deep, or infected pressure injuries, particularly when the wound involves tissue necrosis or extends to muscle, bone, or underlying structures, having the goal of removing necrotic tissue, promoting wound healing, and covering the defect to prevent further complications [16].

This study is conducted in an emergency hospital that does not serve as a referral center, focusing instead of on the management and prevention of pressure injuries within the context of acute care and initial treatment. The study aims to evaluate the prevalence, risk factors, and management strategies of pressure injuries in patients admitted to an emergency hospital in order to improve patient outcomes in critical care settings.

## 2. Material and Methods

We conducted a retrospective study including the clinical records of patients with pressure injuries admitted to the Emergency Clinical Hospital over a 2 year period, between 1 September 2022 and 31 August 2024.

This paper was planned in compliance with the Patient Rights Directive and ethical rules defined by the principles of the Declaration of Helsinki. We first obtained the approval and afterward started collecting and analyzing the data from medical records. This is a retrospective study and was approved by the Hospital Ethics Committee of Bucharest Emergency Clinical Hospital (approval number 2582, approval date: 26 March 2024).

The information retrieved from the medical records referred to sex, age, comorbidities, nutrition status, blood tests (including hemoglobin, serum albumin, and proteins), length of stay, ICU stay, grading and localization of pressure ulcers, microorganisms identified from the pressure ulcers and the surgical treatment. The obtained data were recorded in a Microsoft Excel database as either numerical or categorical variables. Descriptive and inferential statistical methods were employed for data analysis and presentation. Continuous variables are summarized as mean ± standard deviation to represent central tendency and variability, while categorical variables are expressed as frequencies to illustrate their distribution. The mean, median, standard deviation, and confidence interval values were calculated where suitable.

**Figure 1:**
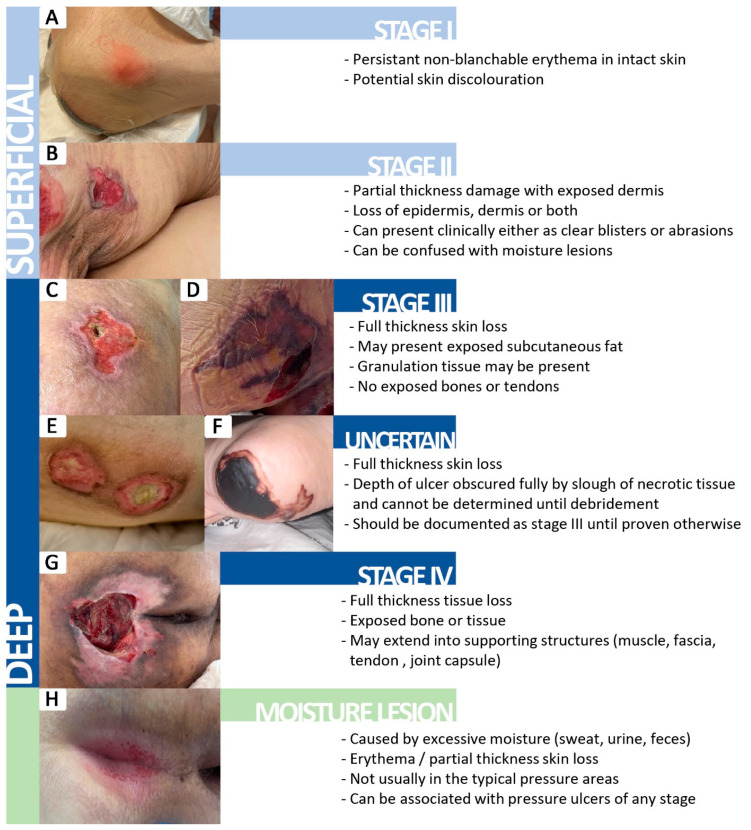
The clinical appearance of different stages of pressure injuries based on the NPUAP consensus [17]. (**A**) Stage I pressure injury; (**B**) Stage II pressure injury; (**C**,**D**) Stage III pressure injury; (**E**,**F**) Uncertain stage; (**G**) Stage IV pressure injury; (**H**) Moisture lesion.

We included in this study patients examined and treated by the plastic surgery team who associated pressure injuries at the moment of hospital admission or patients who developed pressure ulcers during their hospital stay. Patients with incomplete medical documentation and patients without plastic surgery evaluation were excluded from the study. Pressure sores identified in the study were classified in our clinic according to the latest National Pressure Ulcer Advisory Panel (NPUAP) consensus (Figure 1) [17]. Stage 1 pressure injury is represented by intact skin with a localized area of nonblanchable erythema. Stage 2 pressure injury is a partial-thickness skin loss with exposed dermis. In stage 3, pressure injury is a full-thickness skin loss, in which adipose (fat) is visible in the ulcer and granulation tissue is often present, and stage 4 is a full-thickness skin and tissue loss with exposed or directly palpable fascia, muscle, tendon, ligament, cartilage, or bone in the ulcer. Undermining, tunneling, and epibole (rolled edges) may be present. Stage 4 represents the most severe form of pressure injury, often requiring surgical intervention. In addition to the previously mentioned stages, the NPUAP consensus described the unstageable pressure injury and the deep tissue pressure injury. Unstageable pressure injury occurs when the extent of tissue damage cannot be determined because the wound is obscured by slough or eschar (dead tissue). Once the slough or eschar is removed, the injury may reveal a stage 3 or stage 4 pressure injury. A deep tissue pressure injury is marked by persistent nonblanchable deep red, maroon, or purple discoloration of the skin, often observed in intact or nonintact skin. This indicates damage to underlying tissue, even if the skin remains intact. The injury may progress rapidly or evolve into a deeper tissue wound [14].

Microbiological testing was performed on all patients. If the clinical appearance and paraclinical findings suggest infection, we used prelevation swabs after thorough lavage and superficial debridement to obtain relevant data regarding the status of deeper tissues. One challenge in our hospital is that tissue culture results typically take about a week to process. In patients with pressure injuries presenting with clinical signs of infection, since swab results are available more quickly, antibiotic therapy was adjusted promptly based on these preliminary results, allowing for timely intervention and reducing the risk of septic complications, especially in critical patients. In patients without clinical signs of infection, we refrained from initiating antibiotic therapy solely based on swab results. While swabs can provide valuable information, they may not always reflect an active infection, as they can detect colonization rather than true infection. Additionally, when surgical debridement was performed, soft-tissue samples were systematically collected. In patients with grade 4 pressure ulcers, where there was exposure of bone during debridement, both bone and soft tissue samples were obtained. This was undertaken to accurately assess the extent of infection and tissue involvement.

The samples were sent to the microbiology laboratory for bacterial and fungal cultures. Standard culturing techniques, including aerobic and anaerobic incubation, were used to identify the microorganisms present in the pressure ulcers. Antimicrobial susceptibility testing was performed for the isolated pathogens to assess the presence of antimicrobial resistance. Multidrug-resistant bacteria (MDR) were defined as bacteria with acquired resistance to at least one agent in three or more antimicrobial categories, while extensively drug-resistant bacteria (XDR) were defined as bacteria that remain susceptible to only one or two antimicrobial categories, according to the standardized international terminology established in the consensus from European Centre for Disease Prevention and Control (ECDC) and the Centers for Disease Control and Prevention (CDC) [18].

The strategies implemented to prevent bed sores prior to diagnosis, as well as the measures taken to limit the progression of pressure injuries once they developed, were not included in the study. As a result, it was not possible to correlate the data on the care provided to individual patients with their wound development or healing outcomes. The standard recommendations for all patients with pressure sores in our clinic include active and passive mobilization every 2 h, avoiding pressure on the affected area, correcting protein malnutrition and anemia, rigorous physical hygiene, and maintaining the skin clean and hydrated, but without local moisture.

The patients included in the study were admitted to our hospital for a single instance of care, as it is an emergency tertiary hospital. Given the nature of our institution, which is focused on providing immediate care rather than long-term management, the patients were referred to territorial hospitals for follow-up care. Since follow-up care was managed outside of our facility, there was no centralized system to monitor the ongoing progress of patients.

## 3. Results

A total of 176 patients with clinically diagnosed pressure ulcers were included in the study. Of the 176 patients enrolled in the study, 105 patients (59.66%) had only one pressure ulcer, while 71 patients (40.34%) had multiple pressure ulcers. We recorded a total of 298 pressure injuries, with the average number of pressure ulcers per patient being 1.69. Among the 298 bedsores identified, 10.74% were grade I, 25.17% grade II, 28.52% grade III, and 35.57% of bedsores were grade IV pressure injuries (Table 1).

Of all the 176 patients, 131 patients (74.43%) had deep pressure injuries, either a stage IV ulcer, a stage III ulcer, or an association of the 2 stages, meaning we identified 79 patients (44.89%) that had at least 1 stage IV ulcer and 52 patients (29.55%) with stage III pressure ulcers. The distribution of pressure injuries is shown in Table 1, with the most common sites being the sacrum (40.94%), ischium (15.1%), heel (14.43%), and greater trochanter (8.39%) (Table 1).

The median age of the patients in our study was 76 years. Among them, 83 patients (47.15%) were between 60 and 80 years old and 65 patients (36.93%) were ≥80 years old (Table 1).

**Figure 2:**
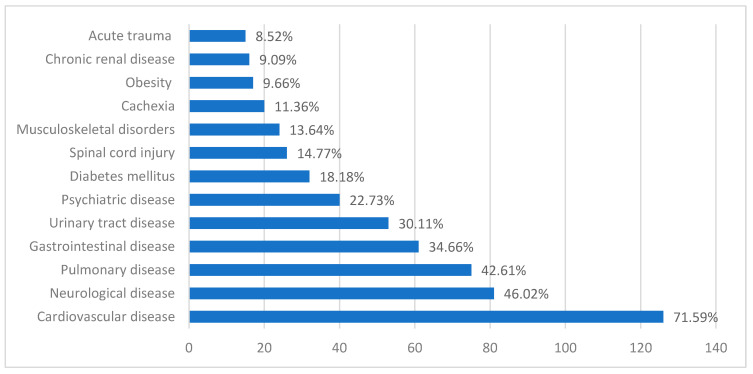
Distribution of comorbidities among the study group. Obesity is defined by a BMI of over 30 kg/m^2^; cachexia is defined by a BMI of under 18.5 that associates significant weight loss in the prior 6 months, loss of lean muscular mass, metabolic disturbances, or decrease of physical performance; diabetes mellitus is defined by a preexisting diagnosis made by two separate fasting blood sugar levels that exceed 126 mg/dL; cardiovascular diseases such as arterial hypertension, heart arrhythmias, atherosclerotic heart disease, congestive heart failure, heart valve disease, and structural heart disease; pulmonary diseases such as chronic obstructive pulmonary disease, asthma, pulmonary fibrosis, pulmonary hypertension, bronchiectasis, pulmonary infections, and lung cancer; gastrointestinal diseases include previous gastrointestinal surgeries, gastritis, ulcers, inflammatory bowel diseases, liver diseases, pancreatic disorders, and gallbladder disease; chronic renal disease such as chronic renal disease, dialysis in end-stage renal disease, and renal transplant; urinary tract diseases include recurrent nephrolithiasis, urinary tract infections, prostate hyperplasia, and urinary incontinence; neurological disease such as history of stroke, dementia, Alzheimer’s disease, epilepsy, peripheral neuropathies; psychiatric diseases such as depressive disorders, anxiety disorders, substance use disorders, schizophrenia, psychotic episodes, or delirium; chronic locomotor system disease such as degenerative joint disease, osteoporosis, and previous orthopedic treatment sequelae; spinal cord injury as defined by movement impairment in patients with posttraumatic injuries to the spinal cord; acute trauma such as polytrauma, high-impact motor vehicle collisions, and falls from significant heights in the acute setting at the moment of consult.

In our study group, patients had several associated health issues, with the distribution shown in Figure 2.

**Figure 3:**
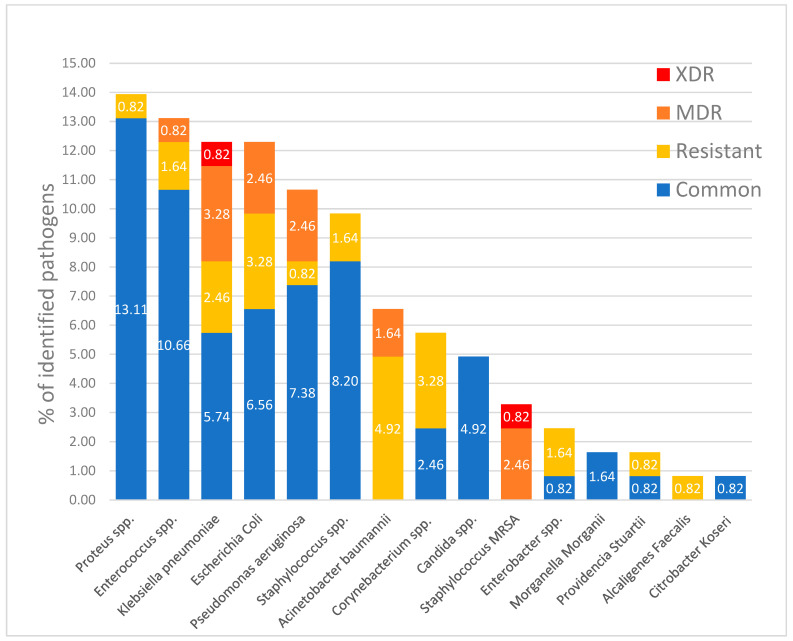
Proportion of microorganisms identified in positive samples from the pressure injuries, including resistant, MDR, and XDR bacteria.

The most common microorganisms identified in pressure ulcers in our study group were *Proteus* spp. in 17 patients (9.65% of patients), *Enterococcus* spp. in 16 patients (9.09% of patients), *Klebsiella pneumonia* and *Escherichia coli* and in 15 patients (8.52% of patients), with the distribution and patterns of resistance shown in Figure 3.

**Figure 4:**
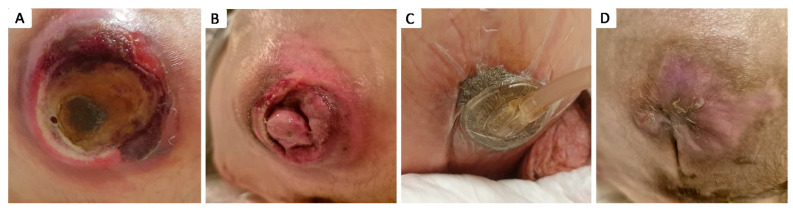
Stage IV 4 × 4 cm ischiatic pressure ulcer in 21 y.o. male with a history of neonatal hypoxic encephalopathy, epilepsy, major neurocognitive disorder, stage IV thoraco-lumbar scoliosis, dysphagia, chronic percutaneous endoscopic gastrostomy, chronic gastritis, and severe cachexy; (**A**) aspect of pressure ulcer prior to debridement; (**B**) aspect of pressure ulcer after debridement, covered by granulation tissue; (**C**) negative pressure wound therapy of the pressure ulcer; (**D**) aspect after negative pressure wound therapy assisted secondary healing of the pressure ulcer.

**Figure 5:**
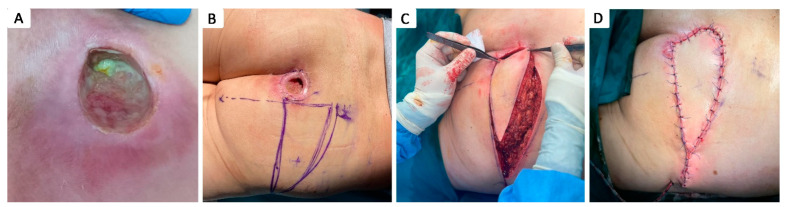
Stage IV 5 × 5 cm sacral pressure ulcer in 63 y.o. male smoker with a history of myocardial infarction and dyslipidemia; (**A**) aspect of sacral pressure ulcer previously treated conservatively, covered with slough, previous to sharp debridement; (**B**) preoperative markings; (**C**) advancement of a unilateral V–Y advancement flap based on a superior gluteal artery perforator; (**D**) postoperative aspect.

**Figure 6:**
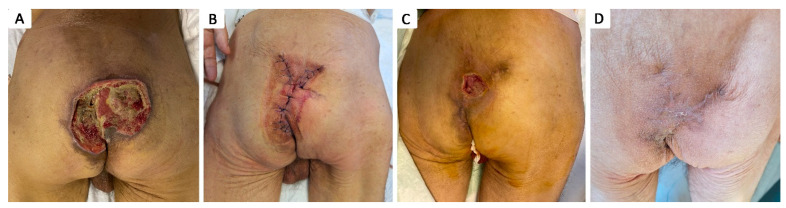
Stage IV 12 × 10 cm sacral pressure ulcer in 62 y.o. with a history of intestinal infarction, subtotal enterectomy, and jejunostomy, subsequent short bowel dysmetabolic syndrome, hepatic abscesses, and cachexia; (**A**) aspect of grade IV sacral pressure ulcer after seriate debridement and negative pressure vacuum wound therapy; (**B**) fourth day postoperative aspect after local cutaneous flap advancement with preservation of all perforator blood vessels encountered during dissection. Negative pressure wound therapy was used on top of the skin suture line to facilitate wound healing; (**C**) three-week postoperative aspect, with superficial 2.5 × 2.5 cm skin ulceration, which was treated conservatively with dressings; (**D**) aspect after healing.

**Figure 7:**
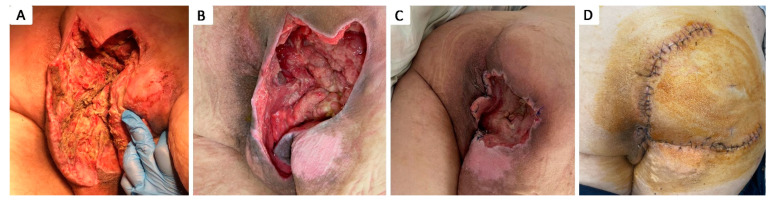
Stage IV 7 × 8 cm therapeutically neglected ischiatic pressure ulcer complicated with Fournier gangrene in 48 y.o. paraplegic male patient with a history of T4–T5 spinal cord injury after accidental fall, stage I obesity, stage III hypertension, long-term urinary catheter use, and repeated catheter-associated urinary infections; (**A**) aspect of necrotic tissue during debridement of Fournier gangrene; (**B**) aspect of ulcer after seriate debridement and infection control; (**C**) aspect of ulcer after negative pressure wound therapy and partial wound closure; (**D**) two-week postoperative aspect after defect coverage with cutaneous superior gluteal artery perforator based flap.

**Figure 8:**
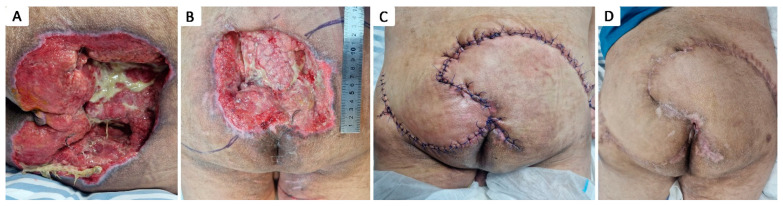
Stage IV 16 × 14 cm sacral pressure ulcer in 54 y.o. paraplegic male with a history of T5 spinal cord injury after accidental fall, multiple rib fractures, pneumothorax, right scapula fracture, and stage III obesity; (**A**) aspect of pressure ulcer, partially covered with slough; (**B**) preoperative aspect; (**C**) immediate postoperative aspect after defect coverage with bilateral gluteal musculocutaneous rotational flaps; (**D**) aspect after healing.

**Figure 9:**
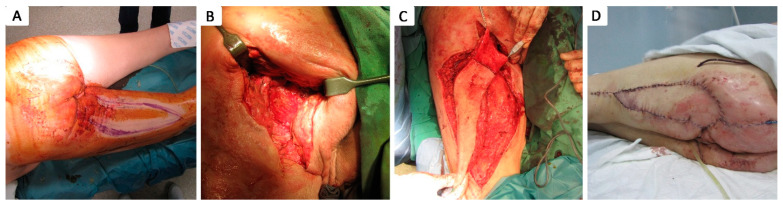
Stage IV 5 × 3 cm deep ischiatic pressure ulcer complicated with ischium osteomyelitis in a 72 y.o. smoker patient with a history of peripheral arteriopathy, chronic peripheral venous insufficiency, and trauma of the hip; (**A**) preoperative aspect; (**B**) aspect of the ischiatic pressure ulcer with exposure of ischium, after debridement and control of infection; (**C**) defect coverage with biceps femoris advancement flap with de-epithelialized flap tip in order to fill the defect cavity with vascularized tissue; (**D**) one-week postoperative aspect.

**Figure 10:**
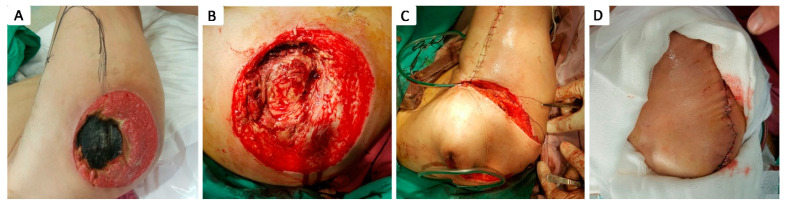
Stage IV 12 × 12 trochanteric pressure ulcer in 40 y.o. female patient with a history of coma induced by abuse of psychoactive substances; (**A**) aspect of pressure ulcer partially covered by necrotic tissue; (**B**) aspect of ulcer after debridement; (**C**) defect coverage with a tensor fascia lata fasciomyocutaneous translational flap; (**D**) postoperative aspect on day 3.

Of the 79 patients with at least one grade 4 pressure ulcer, 17 patients (21.52%) did not undergo surgical debridement due to their critical condition, as their clinical and biological status were considered unsuitable for surgery, and they passed away within the first few days of admission. The other 62 patients with grade 4 pressure ulcers (78.48%) benefited from surgical intervention consisting of necrotic tissue seriate debridement, followed by periodic thorough antiseptic lavage and exudate-absorbing dressings or negative pressure vacuum therapy, either to assist in secondary healing or as an intermediate treatment until definitive surgical coverage was achieved. Closure during hospital stay was achieved by surgical intervention in 14 cases (17.72%): 2 by secondary healing following negative pressure vacuum therapy (Figure 4), 1 by direct suture following negative pressure vacuum therapy, 3 by direct suture following seriate debridement, 1 by skin grafting, and 7 by local advancement or rotation flaps (Figure 5, Figure 6, Figure 7, Figure 8, Figure 9 and Figure 10). Among the patients who underwent flap coverage, we recorded three cases of minor wound dehiscence, which healed by secondary healing.

Of the 52 patients with grade 3 pressure injuries, 25 patients (48.08%) were deemed unsuitable for surgery and died in the first 7 days after admission, while 27 patients (51.92%) benefited from surgical intervention consisting in necrotic tissue seriate debridement, followed by periodic thorough antiseptic lavage and exudate-absorbing dressings, or negative pressure vacuum therapy in order to assist in secondary healing.

The decision to choose closure versus sole debridement and vacuum-assisted closure was based on the patient’s general condition and comorbidities, the biological status, and the possibility of positioning the patient favorably after surgery, avoiding pressure on the operated area. In cases of deep pressure sores, the goal was to achieve coverage after thorough debridement and ensuring negative cultures from the wounds, with the aim of promoting quicker healing through flap surgery. The negative pressure vacuum therapy was applied temporarily as part of this approach. Notably, in three cases, the use of vacuum therapy led to favorable outcomes, and no further coverage procedures were needed.

After flap surgery, we typically waited 14–21 days to avoid placing pressure on the surgical site until the wounds were fully healed. If healing was satisfactory within one month, we allowed patients to bear weight on the operated area, but with precautions. For patients who have had ischial pressure ulcers, we recommended limiting time spent sitting and encouraged short periods of seating while alternating with regular position changes.

Among the patients who underwent flap coverage, we recorded three cases of minor wound dehiscence, which healed by secondary healing.

## 4. Discussion

Pressure ulcers affect patients in physical, social, and psychological ways due to complications such as pain, local and systemic infections, increased care needs, prolonged rehabilitation, the requirement for bed rest, and frequent hospitalization, which are a burden for the healthcare systems worldwide [19,20]. Research has shown that the health-related quality of life of individuals with pressure ulcers is lower than that of similar individuals without pressure ulcers [21].

This study was conducted in an emergency hospital whose focus is on providing treatment in acute settings. The plastic surgery team was primarily called upon for cases of deep pressure ulcers, which explains the differing proportion of pressure ulcers compared to data found in the literature. As the hospital is not a referral center for pressure ulcers, these were either documented as present upon patient admission, particularly in elderly or bedridden patients, often due to the worsening of underlying pathologies, or developed during hospitalization in patients with multiple risk factors for pressure ulcer formation, and occasionally, patients were referred to other specialized care centers. In our study, we identified stage III and IV pressure injuries more frequently. Among the 298 bedsores identified, 10.74% were grade I, 25.17% grade II, 28.52% grade III, and 35.57% of bedsores were grade IV pressure injuries. A systematic review and meta-analysis regarding the incidence of pressure injuries in hospitalized adult patients showed that superficial pressure injuries (stage I and II) are the most frequent stages, with stage I pressure injuries representing 43.5% of the registered cases and stage II pressure injuries representing 28.0% of the registered cases [22]. The most common sites for pressure injuries in our study were the sacrum (40.94%), ischium (15.1%), heel (14.43%), and greater trochanter (8.39%). Other studies also found as common sites for pressure ulcers were the sacrum, followed by ischium, heels, and trochanters [23,24].

The median age of the patients in our study was 76 years. Overall, 83 patients (47.15%) were between 60 and 80 years old, and 65 patients (36.93%) were ≥80 years old. In this group of elderly patients, comorbidities were common, and pressure ulcers were either present upon admission in patients who were previously immobilized in bed or developed during the hospitalization in the context of the worsening of underlying pathologies, in sedated, ventilated, and/or bedridden patients, especially in the ICU. Mortality in the study group was 36.93%, and 23.3% of patients died in the first 7 days. In our study, mortality was due to the patient’s poor condition, with pressure ulcers being a consequence of their clinical and biological deterioration rather than being caused directly by the pressure ulcers themselves.

In our study, we identified several risk factors for the development of pressure sores in our patients. Among them, 14.7% had spinal cord injury, and 46.02% had other neurological diseases such as stroke, Alzheimer’s disease, or dementia. Nutritional deficiency was frequent in our patients, with cachexia being present in 11.36% of the patients. The mean value of serum albumin was 2.56 g/dL; 95% CI (2.43; 2.69), and the mean value of serum proteins was 5.54 g/dL; 95% CI (5.34; 5.73). The mean value hemoglobin in our study group was 10.43g/dL; 95% CI (10.04; 10.82). Patients in our study benefited from physical therapy, active and passive mobilization every 2 h, specialized pressure-relieving mattresses and cushions, avoiding pressure on the areas with pressure ulcers, and rigorous physical hygiene. The administration of intravenous albumin in pressure ulcers was used to address severe hypoalbuminemia, which can occur due to malnutrition or chronic illness, and a high-protein diet was used when possible. Blood transfusions were only used in cases of moderate to severe anemia.

Serum albumin and hemoglobin levels are frequently used as indicators of nutritional status [25]. A study by Sung et al. showed that in managing patients with pressure injuries in an acute care setting, healing was enhanced by administering protein supplements to maintain serum albumin levels above 2.8 g/dL [26]. Recommendations in patients with pressure ulcers include maintaining a daily calorie intake of 30–35 kcal/kg body weight and 1.25–1.5 g of protein/kg body weight [27]. In a study by Serra et al., intravenous albumin supplementation using 25 g human albumin for 3 days within the first week of admission in the intensive care unit significantly reduced the onset of bedsores [28]. Due to the high cost of albumin and its lack of essential amino acids, such as tryptophan, it is generally not used to correct nutritional status and is instead reserved for treating severe cases of hypoalbuminemia [29].

Patients with pressure injuries show mild anemia (serum hemoglobin 9–11 g/dL) in 70% of cases and moderate anemia in the remaining 30% of cases (Hb 7–8 g/dL) and belong to the group of anemias of chronic disorders associated with a proinflammatory status [29]. Hemoglobin levels under 10 g/dL have been previously associated with an increased risk for pressure injuries because low hemoglobin levels result in reduced oxygen delivery to tissues, which impairs wound healing and increases tissue susceptibility to damage [30]. A study by Keast et al. on patients with stage 4 pressure ulcers who received human recombinant erythropoietin for 6 weeks to treat anemia of chronic disease found that all patients experienced not only an increase in hemoglobin levels but also an improvement in the condition of their ulcers. Specifically, ulcer depth decreased, wound surface area reduced, and the appearance of the wound bed as well as the extent of undermining, improved in every ulcer [31]. For patients with anemia of chronic disease, other supplements, such as intravenous iron supplementation vitamin B12 and folate, may also be used if deficiencies in these nutrients are present [32]. Blood transfusions may be useful in severe cases of anemia [33].

Pressure ulcers are commonly associated with several comorbidities that can increase the risk of their development and complicate healing. Diabetes mellitus is strongly linked to a higher incidence of pressure ulcers, as it impairs circulation, reduces sensation, and delays wound healing. Other conditions such as obesity and hypertension also contribute to the risk, with excess weight increasing pressure on vulnerable areas and high blood pressure compromising circulation [34,35,36]. Accordingly, in our study group, 71.59% of patients with pressure sores had cardiovascular disease, 18.18% of them had diabetes mellitus, and 9.66% of patients had obesity.

The key questions for a plastic surgeon to consider are how pressure ulcers should be managed and which patients would benefit from surgical intervention. Superficial pressure ulcers (stages I and II) typically respond well to conservative treatments. However, more severe ulcers (stages III and IV), often seen in patients with spinal cord injuries, frequently necessitate surgical intervention [37,38]. The goal of the surgery should be improving the patient’s overall condition, and the most suitable surgical method may not always be the most apparent choice. Success depends on the availability of resources at all levels, a multidisciplinary approach including surgeon, internist, dietitians, dedicated and well-trained nurses, primary care physicians, and nursing home caregivers, because without these, surgery is likely to fail due to inadequate postoperative care and support, increasing the likelihood of ulcer recurrence [37,39,40].

The core elements of pressure ulcer treatment include relieving pressure from the affected area, removing dead or damaged tissue through debridement, ensuring proper drainage of infections, and maintaining consistent wound care to facilitate healing [5]. A physiological response to tissue injuries follows three major phases of wound healing: inflammation, proliferation, and remodeling. Unfortunately, in the context of pressure ulcers, the wound-healing process could be arrested or impeded by multiple factors. Management of pressure ulcers focuses on promoting natural wound healing by relieving pressure, performing debridement, managing colonization or infection, providing nutritional support, and implementing strategies to prevent recurrence [4]. Pain and discomfort should be controlled by analgesics. Whenever possible, the patient should be moved from the bed to a chair, and physical therapy should be consulted for assistance with ambulation and/or exercise [41]. Prolonged bed rest can lead to a range of other serious complications. Immobility causes muscle weakness and atrophy, especially in the legs and back. This can impair mobility and make recovery more difficult once the person is able to move again [42,43]. Additionally, joint stiffness and the development of contractures can occur, further limiting the range of motion [44]. Another major concern is the development of deep vein thrombosis (DVT), as immobility slows blood circulation and can lead to the formation of blood clots, especially in the lower extremities [45,46].

In our study group, microbiological testing using swabs and soft-tissue samples was performed on all patients, and antibiotic therapy was initiated only when clinical signs of infection were present. In cases of grade 4 pressure ulcers with bone exposure, both bone and soft tissue samples were collected during debridement to assess the extent of infection, and antibiotic therapy was tailored accordingly. Bacterial infections are the most common complication of pressure ulcers and can lead to cellulitis, abscesses, bursitis, and osteomyelitis [47]. The diagnosis of infection in pressure ulcers is mostly clinical, as superficial specimens are almost always positive for bacterial contamination; therefore, culture swabs will often be unreliable in antibiotic targeting. Other sampling techniques include needle aspiration, soft tissue biopsy, or bone biopsy. It is considered that deeper sampling is as informative as it gets and can be used to target antibiotic therapy in patients with positive clinical diagnoses of pressure ulcer infection. Even so, the diagnosis of infection should not be based solely on a single deep tissue biopsy in a pressure ulcer without clinical suspicion of infection, as the samples can often be contaminated, and repeated tissue samples are often recommended. Therefore, multiple deep tissue sampling is recommended and should only be performed in the case of clinical infection suspicion, before antimicrobial prescription, and after proper wound cleaning and debridement [48,49]. Diagnosis of osteomyelitis in pressure ulcers has proven to be difficult and imprecise because of the low rate of sensitivity and specificity of imagistic investigations. Magnetic resonance imaging can be used for evaluating osteomyelitis in non-healing wounds, but it has been described as inefficient in discerning between healed osteomyelitis and active infection, and is currently not routinely used for diagnosis [50]. Imaging techniques often fail in differentiating between osteomyelitis and long-term structural pressure changes in bones, but are useful tools for guiding biopsies [51]. Bone biopsies are being used as a standard point of reference in osteomyelitis diagnosis, but inconsistencies between histological and microbiological studies have been described [52,53]. Anaerobic cultures are not always used due to the increased difficulty of sample harvest, deposit, transport, and culture; therefore, information regarding anaerobic pathogens is likely less represented in published studies. Coupled with the fact that culture results can also be affected by antibiotic therapy administered prior to sample harvest, which sometimes cannot be avoided in the clinical setting, the effect of these phenomena may impact the results of current literature [54,55].

According to available literature, ESKAPE pathogens account for most bacterial surface colonization, alongside coagulase-negative staphylococci and *Proteus* spp. [56,57]. In pressure ulcer-associated osteomyelitis, the most frequent bacteria encountered were Staphylococcus aureus and Enterobacteriaceae, followed by Peptostreptococcus and Bacteroides species [52,58,59,60]. The most frequent species we identified in our study group were *Proteus* spp. (9.65% of patients), *Enterococcus* spp. (9.09% of patients), *Klebsiella pneumonia* (8.52% of patients), and *Escherichia coli* (8.52% of patients).

Of the 176 patients enrolled in the study, 105 patients (59.66%) had only one pressure ulcer, while 71 patients (40.34%) had multiple pressure ulcers. The average number of pressure ulcers per patient was 1.69, with a median of 1. Additionally, 79 patients (44.89%) had at least one grade 4 pressure ulcer.

Of the 131 patients with deep pressure injuries, 89 patients (67.94%) benefited from surgical sharp debridement. Several factors were identified in patients with deep pressure ulcers where debridement was not performed. These included a short time between hospital admission and death, ongoing hemodynamic instability, and critically low levels of platelets and serum hemoglobin that led to the delay in surgical treatment due to the need to correct anemia and thrombocytopenia first. Thrombocytopenia affects approximately 35% to 40% of patients in intensive care units, with sepsis being the leading cause, contributing to an incidence rate of over 50% in critically ill patients. Since thrombocytopenia increases the risk of bleeding, it should be corrected prior to surgery [61,62,63]. Additionally, surgery was not performed in the case of patients who lacked the necessary conditions for postoperative management, which would have led to postoperative complications and morbidity at the donor site, especially in patients with acute trauma who could not be properly positioned after surgery. In the case of critically ill patients, the benefits were carefully weighed, taking into account their overall condition and the potential risks associated with surgical intervention. Besides, elderly patients tended to refuse surgical treatment and opted for conservative management instead, especially in the case of patients with less extensive pressure injuries without bone exposure.

The majority of patients with deep pressure injuries in our study benefited from surgical intervention consisting of necrotic tissue seriate debridement, followed by periodic thorough antiseptic lavage and exudate-absorbing dressings, or negative pressure vacuum therapy, depending on the level of wound fluid secretion and wound bacterial contamination.

Negative pressure wound therapy (NPWT), or vacuum-assisted closure (VAC), has become a widely recognized and effective treatment for managing complex wounds, particularly in the context of chronic wounds and infections [64]. This therapeutic modality uses controlled negative pressure to promote wound healing through a combination of mechanical and molecular mechanisms. The mechanical force applied by the negative pressure helps to contract the wound edges, while at the molecular level, NPWT stimulates the secretion of anti-inflammatory and angiogenic growth factors, such as interleukin-10 and vascular endothelial growth factor (VEGF), involved in promoting tissue regeneration, supporting the formation of new blood vessels (neo-angiogenesis), and enhancing epithelialization [65,66,67]. One of the primary indications of NPWT in pressure sores is for stage 3 or 4 decubitus ulcers in the absence of malignancy, active soft tissue infection, untreated underlying osteomyelitis, unexplored fistulas, known allergies to acrylic adhesives or exposed blood vessels, organs, or nerves [68]. Due to insufficient critical data on complete wound healing, adverse events, healing time, and cost-effectiveness, the effectiveness, safety, and acceptability of NPWT for treating pressure ulcers compared to standard care remain uncertain. However, NPWT may offer benefits over usual care by accelerating the reduction of pressure ulcer size and severity, lessening pain, and decreasing the frequency of dressing changes [69].

In the majority of cases of deep pressure injuries in our study, the surgical management involved multiple steps consisting of seriate debridement with or without the use of negative pressure vacuum therapy, followed by surgical wound closure or secondary healing. In two cases, the surgical debridement and coverage of the pressure ulcer were performed in one step. Of the 79 patients with stage IV pressure ulcers, closure was achieved by surgical intervention in 14 cases (17.72%): 2 by secondary healing following negative pressure vacuum therapy, 1 by direct suture following negative pressure vacuum therapy, 3 by direct suture following seriate debridement, 1 by skin grafting and 7 by local advancement or rotation flaps.

In our study, we did not perform any colostomies on our patients. While colostomy may be considered in certain severe cases of pressure ulcers, the potential complications, including infection, stoma-related issues, and the patient’s overall health status, led us to avoid complex auxiliary surgical procedures. Instead, we focused on more conservative management approaches, prioritizing rigorous hygiene and wound care. In four cases, we used fecal collector systems for a short period following coverage surgery. This was undertaken to protect the surgical site and prevent contamination from fecal matter, allowing the wound to heal without further irritation or infection.

Surgical treatment of pressure ulcers is reserved for grade III and IV pressure ulcers refractory to conservative treatment [70]. It consists of debridement and coverage of the defect. Thorough debridement is required before reconstruction in order to achieve a clean defect lined with healthy, bleeding tissue suitable for coverage. In cases with stable, clean, low-exuding pressure ulcers in balanced patients, surgical debridement and coverage of the defect can be achieved with a single-staged procedure, although this has proven to rarely be the case in our clinic.

Surgical debridement effectively removes non-viable tissue from chronic pressure wounds, exposing healthy, well-vascularized tissue that supports healing. Various debridement techniques—including autolytic, biological, enzymatic, mechanical, and surgical methods—should be tailored to the wound’s characteristics, considering patient comorbidities, comfort, and preferences [71,72,73]. Debridement is crucial for preparing the wound bed for re-epithelialization by eliminating devitalized tissue, which can nourish bacteria and hinder healing by blocking topical treatments [74].

While surgical debridement is beneficial, it carries risks like bleeding and potential anesthesia complications [75]. During the procedure, tissue samples should be sent for bacterial analysis and histology, as superficial swabs are often unreliable [76]. Methylene blue can be used to trace sinus tracts, aiding in thorough debridement [77].

Potential closure procedures following surgical debridement include any procedure from primary wound closure and skin grafts to more novel approaches such as perforator-based flaps and free tissue transfers. Guidelines on the prevention and management of pressure ulcers make no specific suggestion in regard to the reconstructive options. Given the complexity of the comorbidities associated with the often bedridden pressure ulcer patient and the exhaustive postoperative care regimen, it has proven difficult to establish a standardized surgical conduct for the treatment of this pathology [77,78,79].

Given the lack of data on the frequency of the surgical procedures used in the reconstruction of pressure ulcers, current decisions to employ reconstructive surgery are likely based on local care pathways, the expertise of local surgeons, patient and healthcare professional preferences, and cost considerations [70].

The chosen surgical technique should be adapted to the patient’s needs and underlying conditions. Given the high rate of recurrence of pressure ulcers regardless of the chosen procedure, careful consideration should be given to preserving as much surrounding tissue as possible as a back-up for potential future recurrence of pressure ulcers [80,81,82].

Large musculocutaneous or fasciocutaneous flaps have been described to be more useful for covering pressure ulcers given the ability to fill dead spaces by using the bulk of the flap or even de-epithelialized areas of the flap, reduce risk of seroma formation, provide a tension-free closure with a suture line placed further away from the pressure zone and even by ensuring thicker padding to the reconstructed pressure-prone area [77,83,84].

Studies showed, however, no statistically significant differences between the rate of complications and recurrence following surgical reconstruction using regional perforator-based flaps as opposed to traditional musculocutaneous or fasciocutaneous flaps [82]. Using musculocutaneous, fasciocutaneous, and perforator flaps to cover pressure sores aligns well with surgical principles for treating these ulcers: they provide sufficient tissue to fill dead space, are well vascularized, and generally allow for tension-free closure. However, there is still no consensus on the necessity of including muscle tissue in the flap. Using muscle tissue to cover pressure sores is not a perfect solution. Muscle tissue is less tolerant to ischemia than skin tissue. Experiments have shown that pressure on musculocutaneous flaps causes hypoxia, leading to muscle necrosis without skin necrosis. Furthermore, the pressure-distributing function of muscle flaps diminishes soon after surgery because the muscle in the transferred flaps undergoes significant atrophy [85]. In non-paralyzed patients, it is essential to preserve muscle function to aid in the recovery of mobility. In paralyzed patients, pressure ulcers are prone to recurrence and often require repeated reconstruction. Utilizing muscle tissue as the initial reconstruction method can limit future options for using fasciocutaneous flaps [86].

**Figure 11:**
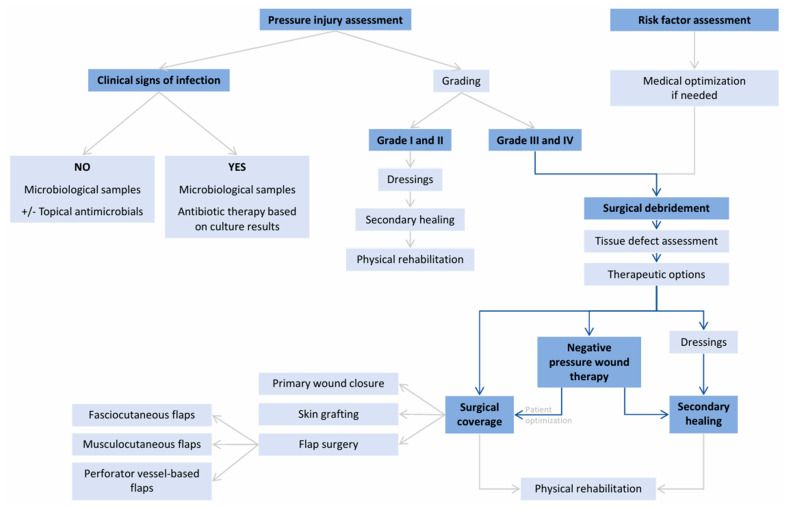
Treatment algorithm for pressure injuries [17,87,88,89,90,91].

A comprehensive algorithm outlining the workflow in the management of pressure injuries is shown in Figure 11.

The prognosis of patients with pressure injuries is closely linked to their ambulatory status. In terms of quality of life, aside from traumatic pathologies that result in lasting sequelae, individuals recovering from pressure ulcers may experience significant discomfort due to the formation of fibrotic, painful scars, which can further be managed through scar remodeling techniques. The application of lipofilling aids in the secondary prevention of recurrence in patients with previous pressure injuries, not only by facilitating volumetric remodeling of the affected area through thickening of the subcutaneous fat layer and increasing the extent of cushioning over the underlying bony prominences but also by changing the microenvironment through the addition of multipotent cells and stimulating factors [92,93,94,95]. In cases of persistent or vicious scarring, additional surgical interventions may be necessary.

A key limitation of the study is its monocentric nature, as it was conducted in a single emergency hospital rather than a specialized reference center. This could potentially limit the generalizability of the findings, as the outcomes observed may not fully reflect those of larger, more specialized institutions. Additionally, the lack of follow-up data, as previously mentioned, represents another significant limitation. Without long-term follow-up, it is difficult to assess the interventions’ effectiveness and any potential complications that may arise after the initial treatment.

## 5. Conclusions

Pressure injuries represent a significant challenge and place a heavy strain on healthcare systems due to prolonged treatment, complex care needs, increased risk of complications, and associated costs. While patients with superficial pressure injuries generally respond well to conservative measures, surgical treatment is reserved for deep pressure ulcers presenting with extensive tissue necrosis or bone osteomyelitis. The decision to proceed with surgery also considers the patient’s general status and comorbidities, the ability to tolerate operative and postoperative care, and the presence of suitable local or regional tissue for durable coverage. Implementing a structured therapeutic algorithm for these patients greatly streamlines the decision-making process, ensuring that clinicians can more systematically identify when to opt for non-surgical treatments and when to proceed to surgical interventions. In this way, the use of a well-defined therapeutic protocol helps reduce uncertainty, increases consistency in care, and ultimately enhances patient outcomes.

## Figures and Tables

**Table 1:** Characteristics of the patients with pressure injuries included in the study.

Age group		20–40		40–60		60–80		80–100		TOTAL	
Number of patients		7		21		83		65		176	
Sex		28.57% F	71.43% M	23.81% F	76.19% M	57.83% F	52.17% M	73.85% F	26.15% M	103 F	73 M
Length of stay (days)		AVG.	MEDIAN	AVG.	MEDIAN	AVG.	MEDIAN	AVG.	MEDIAN	AVG.	MEDIAN
		36	25	31.7	9	17.8	12	11	8	17.81	9.5
ICU admission		Nr.	%	Nr.	%	Nr.	%	Nr.	%	Nr.	%
		3	42.86	6	28.57	24	28.92	11	16.92	44	25
Death		Nr.	%	Nr.	%	Nr.	%	Nr.	%	Nr.	%
		1	14.29%	2	9.52%	33	39.76%	29	44.62%	65	36.93%
Early death (<7 days from admittance)		Nr.	%	Nr.	%	Nr.	%	Nr.	%	Nr.	%
		1	14.29%	1	4.76%	18	21.67%	21	32.31%	41	23.3%
Pressure ulcers		Nr.	%	Nr.	%	Nr.	%	Nr.	%	Nr.	%
	Grade I	4	30.77	3	7.89	14	10.07	11	10.19	32	10.74
	Grade II	1	7.69	13	34.21	33	23.74	28	25.93	75	25.17
	Grade III	0	0	8	21.05	43	30.09	34	31.48	85	28.52
	Grade IV	8	61.54	14	36.84	49	35.25	35	32.41	106	35.57
Pressure ulcers		Nr.	%	Nr.	%	Nr.	%	Nr.	%	Nr.	%
	Sacral	5	38.46	10	26.32	62	44.60	45	41.67	122	40.94
	Ischeatic	1	7.69	10	26.32	20	14.39	14	12.96	45	15.1
	Heel	2	15.38	3	7.89	19	13.67	19	17.59	43	14.43
	Trochanteric	3	23.08	3	7.89	10	7.19	9	8.33	25	8.39
	Malleolus	1	7.69	4	10.53	14	10.07	11	10.19	30	10.07
	Elbow	0	0 0	2	5.26	5	3.60	2	1.85	9	3.02
	Scapular	0	0 0	3	7.89	7	5.04	5	4.63	15	5.03
	Occipital	1	7.69	2	5.26	2	1.44	1	0.93	6	2.01
	Cervical	0	0 0	1	2.63	0	0	2	1.85	3	1
Associated health issues		Nr.	% *	Nr.	% *	Nr.	% *	Nr.	% *	Nr.	% *
	Obesity	1	14.3	2	9.52	12	14.5	2	3.1	17	9.66
	Cachexia	2	28.6	2	9.52	6	7.23	10	15	20	11.36
	Diabetes mellitus	1	14.3	4	19	22	26.5	5	7.7	32	18.18
	Cardiovascular disease	0	0	9	42.9	66	79.5	51	78	126	71.59
	Pulmonary disease	1	14.3	4	19	42	50.6	28	43	75	42.61
	Gastrointestinal disease	3	42.9	5	23.8	31	37.3	22	34	61	34.66
	Chronic renal disease	0	0	1	4.76	6	7.23	9	14	16	9.09
	Urinary tract disease	1	14.3	4	19	23	27.7	25	38	53	30.11
	Neurological disease	4	57.1	5	23.8	42	50.6	30	46	81	46.02
	Psychiatric disease	0	0	3	14.3	17	20.5	20	31	40	22.73
	Chronic locomotor system injuries and deficits	1	14.3	4	19	13	15.7	6	9.2	24	13.64
	Spinal cord injury	3	42.9	4	19	16	19.3	3	4.6	26	14.77
	Acute trauma	0	0	3	14.3	7	8.43	5	7.7	15	2.84

* percentage of patients that associate the condition.

## Data Availability

Data is available upon request from the authors.
